# Contrasting responses of grain yield to reducing nitrogen application rate in double- and single-season rice

**DOI:** 10.1038/s41598-018-36572-0

**Published:** 2019-01-14

**Authors:** Min Huang, Long Fan, Yingbin Zou

**Affiliations:** Southern Regional Collaborative Innovation Center for Grain and Oil Crops (CICGO), Hunan Agricultural University, Changsha, China

## Abstract

Reducing N rate without sacrificing grain yield is crucial for sustainable rice production in China. In this study, field experiments were conducted in 2016 and 2017 to determine whether the response of grain yield to reducing N rate varied between double-season rice (DSR) and single-season rice (SSR). Three N rates were applied for each rice crop, i.e. 150 kg ha^−1^ (N150, the locally recommended N rate), 90 kg ha^−1^ (N90), and 0 kg ha^−1^ (N0). Grain yield was lower under N90 than under N150 in DSR by 11–18%, whereas the difference was not significant in SSR. Grain yield under N0 was 36–63% lower in DSR than in SSR. There was a significant relationship between percentage change in grain yield comparing N90 to N150 with grain yield under N0. Furthermore, it was observed that grain yield under N0 was not significantly associated with growth duration but was closely associated with daily grain yield under N0, and the relationships of daily grain yield under N0 with seasonal average daily mean temperature and solar radiation were not significant. These results indicate that response of grain yield to reducing N rate was more sensitive in DSR compared to SSR due to lower soil N productivity. Growth duration and climatic conditions did not explain the difference in soil N productivity between DSR and SSR. The findings of this study should encourage more research into comparing the inherent traits of plants, especially the morphology and physiology of the root system, between DSR and SSR.

## Introduction

Rice is the staple food crop for about 65% of the population in China^1^. In the past several decades, rice yield has more than tripled in China as a result of the development of new cultivars and improved crop management practices^2^. This increase in rice yield has been important for ensuring food security in China. However, at the same time, fertilizer consumption in China has had an almost linear increase, especially for nitrogen (N) fertilizer^3^. The average N application rate for rice production in China at present is 180 kg ha^−1^, which is about 75% higher than the world average^4,5^. The high N rate has caused substantial environmental costs including enhanced N deposition, soil acidification, and surface water eutrophication^6–8^. Therefore, reducing the N rate without sacrificing grain yield is necessary for sustainable rice production in China.

The capacity of productivity in a soil is referred to as inherent soil productivity and can be expressed by the actual crop yield without fertilizer inputs^9^. Improving soil fertility is an effective way to achieve high inherent soil productivity and consequently reduce the dependence on external fertilizer inputs in rice production^10^. In addition to the soil conditions, the inherent soil productivity also can be affected by the crop. For example, Huang *et al*.^11^ observed that inherent soil N productivity (i.e. grain yield without N application) was increased with the development of new rice cultivars in China.

Hunan province is one of the major rice-producing provinces in China and contributes more than 10% of total rice production (World Rice Statistics, http://ricestat.irri.org:8080/wrs). There are two rice cropping systems in this province: (1) double-season rice (DSR) cropping with early-season rice (ESR) grown from March (sowing) to July (harvesting) and late-season rice (LSR) grown from June to October; and (2) single-season rice (SSR) cropping from April to September. Growth duration is shorter in DSR compared to SSR. This difference in growth duration can result in shorter duration of soil N uptake and consequently may cause lower soil N productivity in DSR than in SSR. Moreover, climatic conditions during growing-seasons are also different between DSR and SSR. In general, seasonal average temperature and solar radiation are lower in DSR than in SSR, because ESR and LSR have lower temperature and solar radiation than SSR during the early and late growth periods, respectively. These differences in temperature and solar radiation may lead to slower crop growth and hence lower soil N productivity in DSR compared to SSR. The potentially different soil N productivity resulted from differences in growth duration, and climatic conditions may further lead to different N responses between DSR and SSR, but no studies have been conducted to confirm and clarify these differences.

In the present study, we compared DSR and SSR under three N rates (recommended, reduced, and zero N rate) in two years. Our objectives were (1) to determine whether the response of grain yield to reducing N rate is different between DSR and SSR, and if so, (2) to identify the factors that contribute to the difference in N response between DSR and SSR.

## Results and Discussion

**Figure 1:**
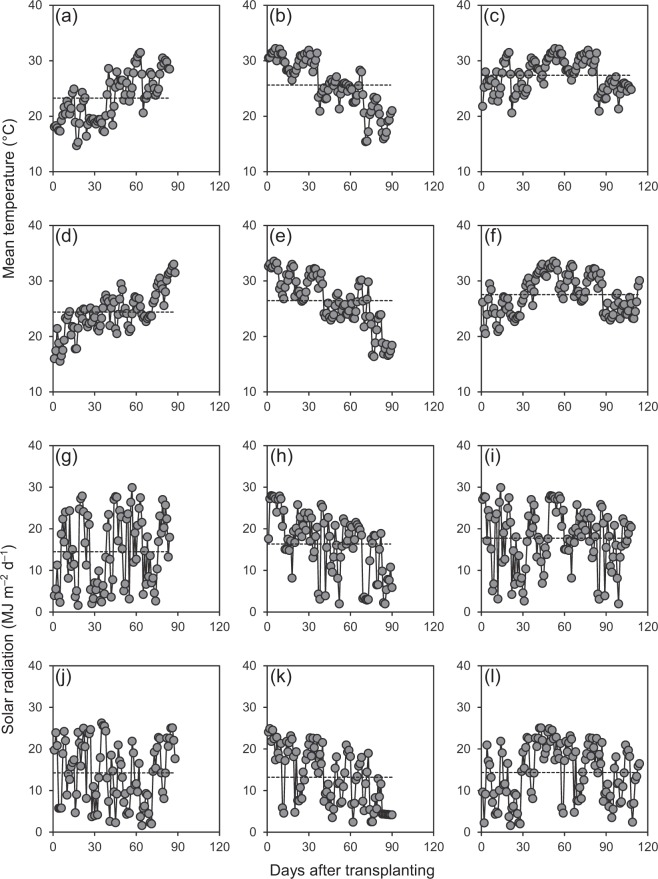
Daily mean temperature (**a**–**f**) and solar radiation (**g**–**l**) during early (**a**,**d**,**g**,**j**), late (**b**,**e**,**h**,**k**) and single rice-growing seasons (**c**,**f**,**i**,**l**) in 2016 (**a**–**c**,**g**–**i**) and 2017 (**d**–**f**,**j**–**l**). Dashed lines represent the seasonal averages.

Average daily mean temperatures during early and late rice-growing seasons were lower than that during the single rice-growing season by 1.8–4.1 °C in 2016 and by 1.1–3.2 °C in 2017 (Fig. 1a–f). In 2016, average daily solar radiation was 8–18% lower during early and late rice-growing seasons than during the single rice-growing season (Fig. 1g–i). In 2017, average daily solar radiation during the early rice-growing season was almost the same as that during the single rice-growing season, while it was 8% lower during the late rice-growing season than during the single rice-growing season (Fig. 1j–l).

**Figure 2:**
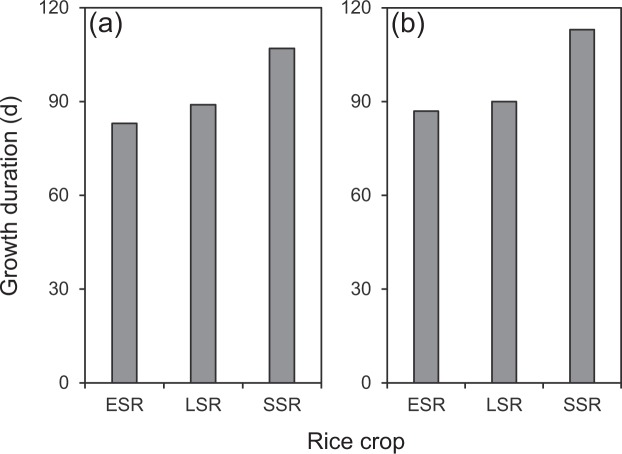
Growth duration from transplanting to maturity in three rice crops in 2016 (**a**) and 2017 (**b**). ESR, LSR and SSR represent early-, late- and single-season rice, respectively.

Growth duration from transplanting to maturity was shorter in ESR and LSR compared to SSR by 18–24 d in 2016 and by 23–26 d in 2017 (Fig. 2a,b). It is well documented that growth duration is affected by temperature in rice^12^. High temperatures shorten the growth duration and vice versa. However, in this study, the shorter growth duration in ESR and LSR than in SSR was not related to temperature because, as mentioned above, the temperature was lower during early and late rice-growing seasons than during the single rice-growing season. Therefore, the difference in growth duration between DSR and SSR should be mainly attributed to different genotypes.

**Figure 3:**
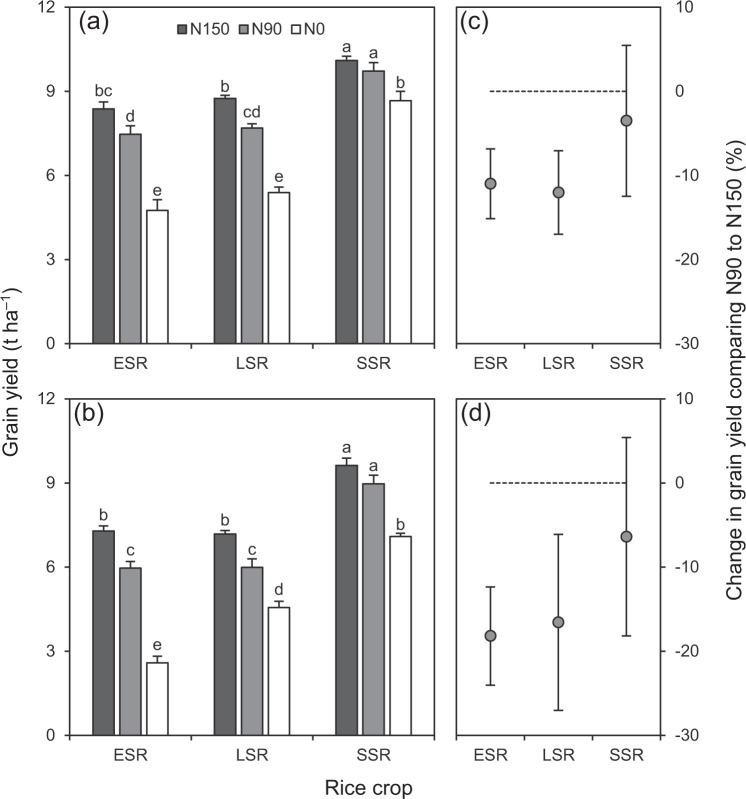
Grain yield under three N rates (**a**,**b**) and percentage change in grain yield comparing N90 to N150 (**c**,**d**) in three rice crops in 2016 (**a**,**c**) and 2017 (**b**,**d**). N150, N90 and N0 represent 150, 90 and 0 kg N ha^−1^, respectively. ESR, LSR and SSR represent early-, late- and single-season rice, respectively. In (**a**,**b**), data are means and SE. Bars with the same letters are not significantly different according to LSD (0.05). In (**c**,**d**), data are means and 95% confidence intervals. Dashed lines are the zero lines.

Grain yield was significantly affected by rice crop, N rate, and their interaction (Table 1). In general, SSR had higher grain yield than ESR and LSR (Fig. 3a,b). Grain yield was reduced by reducing N rate. However, the magnitude of grain yield reduction from reduced N rate (from N150 to N90) varied with the rice crop (Fig. 3a–d). Grain yield was significantly lower under N90 than under N150 in ESR and LSR by 11–12% in 2016 and by 17–18% in 2017, whereas the difference was not significant in SSR in both years.

**Table 1:** *F*-values of analysis of variance for grain yield in three rice crops (early-, late- and single-season rice) under three N rates (150, 90 and 0 kg ha^−1^) in 2016 and 2017.

Source	2016	2017
Rice crop	89.51^***^	160.12^***^
N rate	93.33^***^	148.13^***^
Rice crop × N rate	5.70^**^	7.78^***^

^**^ and^ ***^ denote significance at the 0.01 and 0.001 probability levels, respectively.

**Figure 4:**
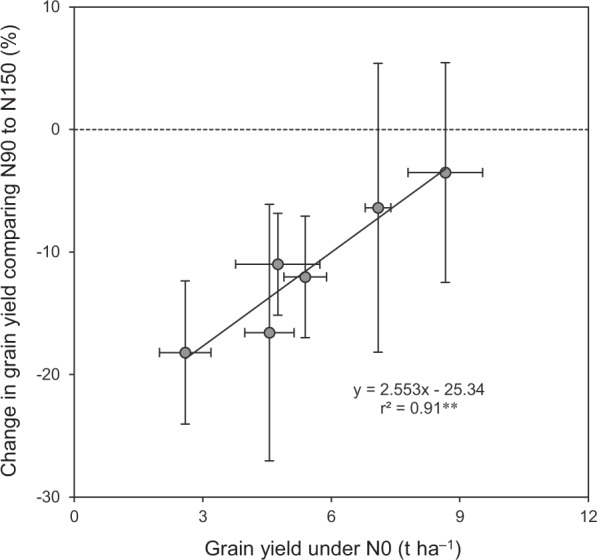
Relationship between percentage change in grain yield comparing N90 to N150 with grain yield under N0 in rice. N150, N90 and N0 represent 150, 90 and 0 kg N ha^−1^, respectively. Data are means and 95% confidence intervals. Dashed line is the zero line. ^**^denotes significance at the 0.01 probability level.

These results indicate that response of grain yield to reducing N rate was more sensitive in DSR (ESR and LSR) compared to SSR. This different N response could be related to the difference in soil N productivity (grain yield under N0) between DSR and SSR, which was significantly lower in ESR and LSR than in SSR by 38–45% in 2016 and by 36–63% in 2017 (Fig. 3a,b). Consistently, a significant relationship was observed between percentage change in grain yield comparing N90 to N150 with grain yield under N0 (Fig. 4). The regression equation of this relationship shows that the percentage change in grain yield comparing N90 to N150 was reduced by about 2.5% for each 1 t ha^−1^ increase in grain yield under N0. These findings support results of previous studies suggesting that improving soil N productivity is an effective way to reduce the dependence of external N inputs in rice production^10,11^. More importantly, the results of this study suggest that the magnitude of the potential of reducing N rate may be smaller in DSR than in SSR due to lower soil N productivity.

**Figure 5:**
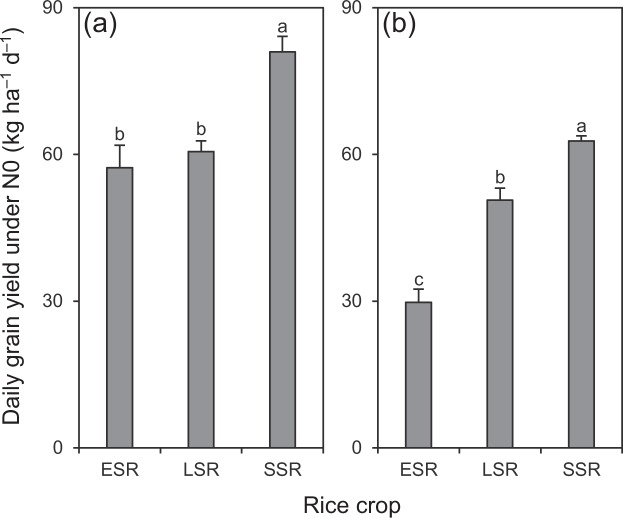
Daily grain yield under N0 in three rice crops in 2016 (**a**) and 2017 (**b**). N0 represents 0 kg N ha^−1^. ESR, LSR and SSR represent early-, late- and single-season rice, respectively. Data are means and SE. Bars with the same letters are not significantly different according to LSD (0.05).

**Figure 6:**
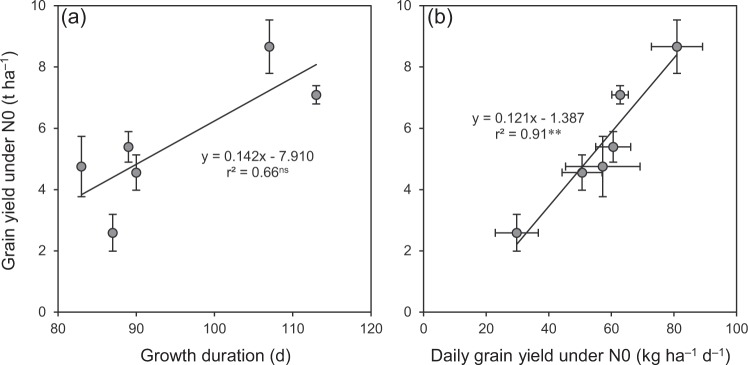
Relationship of grain yield under N0 with growth duration from transplanting to maturity (**a**) and daily grain yield under N0 (**b**) in rice. N0 represents 0 kg N ha^−1^. Data are means and 95% confidence intervals. ^ns^and ^**^denote non-significance at the 0.05 probability level and significance at the 0.01 probability level, respectively.

**Figure 7:**
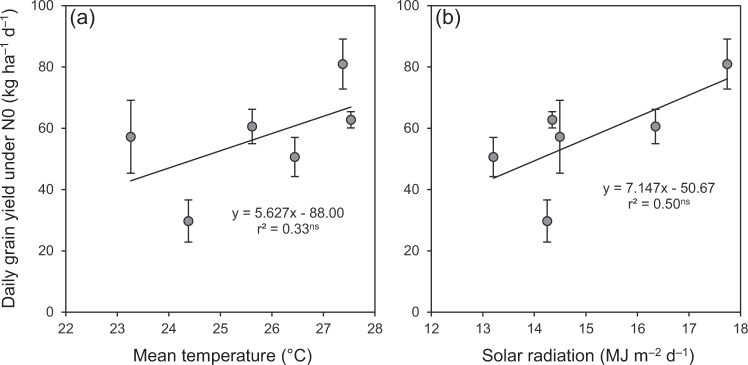
Relationship of daily grain yield under N0 with seasonal average daily mean temperature (**a**) and solar radiation (**b**) in rice. N0 represents 0 kg N ha^−1^. Data are means and 95% confidence intervals. ^ns^ denotes non-significance at the 0.05 probability level.

Similar to the grain yield under N0, daily grain yield under N0 was significantly lower in ESR and LSR than in SSR by 25–29% in 2016 and by 19–53% in 2017 (Fig. 5). More interestingly, grain yield under N0 was not significantly associated with growth duration but was closely associated with daily grain yield under N0 (Fig. 6a,b), and the relationships of daily grain yield under N0 with seasonal average daily mean temperature and solar radiation were not significant (Fig. 7a,b). These observations are not consistent with our initial expectation that the differences in growth duration and climatic conditions may result in different soil N productivity between DSR and SSR. These results also suggest that the different soil N productivity between DSR and SSR might mainly depend on the plant’s inherent traits. In this regard, it is generally accepted that the root system primarily determines the ability of a plant to exploit soil resources^13,14^. The findings of this study should encourage more research into comparing the morphology and physiology of the root system between DSR and SSR. Such research would provide useful information to improve soil N productivity and consequently reduce the dependence of external N inputs in rice, especially for DSR.

Perhaps also interestingly, in contrast to the grain yield under N0, increase in grain yield with N fertilization was generally higher in ESR and LSR than in SSR (Fig. 3a,b). This means that agronomic N use efficiency was higher in DSR than in SSR. One possible reason for this difference might be that the spatial distributions of roots and fertilizer N were better-matched in DSR than in SSR^15,16^, but further studies are required to confirm this hypothesis. In addition, our study has potential limitations that need to be acknowledged. First, the experiment was conducted on only one soil type. Second, only one cultivar was used for each rice crop. Therefore, more experimentation should be done on different soil types with various rice cultivars to obtain broader results.

## Conclusions

DSR had lower soil N productivity than SSR. As a result, response of grain yield to reducing N rate was more sensitive in DSR compared to SSR. Growth duration and climatic conditions did not explain the difference in soil N productivity between DSR and SSR.

## Methods

Field experiments were conducted in a farmer’s field in Yongan Town (28°09′ N, 113°37′ E, 43 m asl), Hunan Province, China, in 2016 and 2017. The soil of the field was a clay type with the following properties: pH = 6.26, organic matter = 44.3 g kg^−1^, total N = 1.50 g kg^−1^, total P = 0.61 g kg^−1^, total K = 7.92 g kg^−1^, NaOH hydrolysable N = 179 mg kg^−1^, NaHCO_3_ extractable P = 16.2 mg kg^−1^, and NH_4_OAc extractable K = 102 mg kg^−1^. The soil characteristics were determined based on samples taken from the 0–20 cm layer, with the methods described by Bao^17^.

Two rice cultivars, Zhongzao 39 and Y-liangyou 1, were used in this study. Zhongzao 39 was grown in early and late rice-growing seasons, while Y-liangyou 1 was grown in the single rice-growing season. These two cultivars were selected because they have been widely grown by rice farmers in the study region. Three N rates were applied to each rice crop (ESR, LSR or SSR), i.e. 150 kg ha^−1^ (N150, the locally recommended N rate), 90 kg ha^−1^ (N90), and 0 kg ha^−1^ (N0). Treatments were arranged in a split-plot design with rice crops (DSR and SSR) as main plots and N rates as subplots. The experiment was replicated six times and subplot size was 20 m^2^.

Rice crops were established according to locally recommended practices. Pre-germinated seeds were sown in a seedbed. For DSR, 25- and 15-day-old seedlings were transplanted on 22 April and 21 July in the early and late seasons, respectively. Transplanting was done at a hill spacing of 20 cm × 16.7 cm with three seedlings per hill. For SSR, 25-day-old seedlings were transplanted on 4 June. Transplanting was done at a hill spacing of 20 cm × 20 cm with two seedlings per hill.

N was applied in three splits: 50% as basal (1 day before transplanting), 30% at early-tillering (7 days after transplanting), and 20% at panicle initiation. Phosphorus (75 kg P_2_O_5_ ha^−1^ as basal) and potassium (75 kg K_2_O ha^−1^ as basal, and 75 kg K_2_O ha^−1^ at panicle initiation) were applied in all subplots. All fertilizers were manually broadcasted and incorporation was made only for basal fertilization. The experimental field was kept flooded (5–10 cm) from transplanting until 7 days before maturity, when the field was drained. Insects and diseases were intensively controlled by chemicals to avoid yield loss.

Daily mean temperature and solar radiation during rice-growing seasons were measured using a Vantage Pro2 automatic weather station (Davis Instruments Corp., Hayward, CA, USA). Growth duration was recorded as number of days from transplanting to maturity. Grain yield was determined from a 5-m^2^ area in each subplot at maturity and adjusted to a moisture content of 14%. Seasonal average daily mean temperature and solar radiation, percentage changes in grain yield comparing N90 to N150, and daily grain yield under N0 (grain yield under N0/growth duration) were calculated.

Statistical analysis was performed in Statistix 8.0 (Analytical Software, Tallahassee, FL, USA). Data were analyzed using analysis of variance and linear regression analysis. Means of treatments were compared based on the least significant difference (LSD) test at the 0.05 probability level.

## Data Availability

All data generated or analysed during this study are included in the article.
